# Establishment of a novel mouse model of renal artery coiling-based chronic hypoperfusion-related kidney injury

**DOI:** 10.1016/j.bbrep.2023.101607

**Published:** 2023-12-15

**Authors:** Yoshimi Imamura-Uehara, Mako Yasuda-Yamahara, Shogo Kuwagata, Kosuke Yamahara, Mamoru Yoshibayashi, Yuki Tanaka-Sasaki, Akio Shimizu, Hisakazu Ogita, Masami Chin-Kanasaki, Shinji Kume

**Affiliations:** a Department of Medicine, Shiga University of Medical Science, Tsukinowa-cho, Otsu, Shiga, 520-2192, Japan; b Division of Molecular Medical Biochemistry, Department of Biochemistry and Molecular Biology, Shiga University of Medical Science, Tsukinowa-cho, Otsu, Shiga, 520-2192, Japan

**Keywords:** Renal hypoperfusion, Chronic ischemia, Artery coiling, Animal model

## Abstract

Renal artery stenosis-induced chronic renal ischemia is an important cause of renal dysfunction, especially in older adults, and its incidence is currently increasing. To elucidate the mechanisms underlying chronic renal hypoperfusion-induced kidney damage, we developed a novel mouse model of renal artery coiling-based chronic hypoperfusion-related kidney injury. This model exhibits decreased renal blood flow and function, atrophy, and parenchymal injury in the coiled kidney, along with compensatory hypertrophy in the non-coiled kidney, without chronic hypertension. The availability of this mouse model, which can develop renal ischemia without genetic modification, will enhance kidney disease research by serving as a new tool to investigate the effects of acquired factors (e.g., obesity and aging) and genetic factors on renal artery stenosis-related renal parenchymal damage.

## Highlights

• A mouse model of chronic renal ischemia was established by renal artery coiling.
• Renal artery coiling caused renal injury without chronic hypertension.
• This model can help to elucidate mechanisms of ischemia-induced renal injury.

## 1 Introduction

The increasing number of patients with end-stage kidney disease (ESKD) requiring dialysis has become a global public health problem, affecting quality of life in those patients and the broader economic aspects of healthcare. Although diabetic nephropathy has been the leading cause of ESKD for many years, research progress concerning diabetes complications has led to the development of new therapeutic strategies, with the potential for improved patient outcomes. In contrast, population aging in many advanced countries has led to growing concern regarding the increasing number of older adults who require dialysis [[1], [2], [3], [4], [5]].

It is estimated that 5%–22 % of older patients with chronic kidney disease have renal artery stenosis, which causes a progressive decline in renal function, along with renal ischemia [[6], [7], [8], [9]]. Therefore, it is important to prevent arterial sclerosis-related renal artery stenosis. However, after stenosis establishment, reversal is challenging; controversy persists regarding whether stenosis relief through vascular intervention leads to the recovery of renal function. Additionally, the renal prognosis in patients with renal artery stenosis is more strongly correlated with the extent of renal parenchymal damage accompanying the stenosis, rather than the degree of stenosis itself. Thus, elucidation of the mechanisms underlying renal artery stenosis-induced renal parenchymal damage should help to reduce the number of patients requiring dialysis to manage ESKD resulting from diseases such as nephrosclerosis, which are increasingly common in aging societies.

The establishment of an appropriate animal model is essential for efforts to understand the pathophysiology of a disease. Existing animal models of renal ischemia include the ischemia-reperfusion model [10,11]. However, this model represents acute kidney injury. Additionally, some mouse models develop atherosclerosis because of severe lipid abnormalities related to deficiencies in ApoE or low-density lipoprotein receptor [12,13]. However, it is difficult to determine whether these kidney injuries are the result of altered lipid metabolism or renal ischemia. In the field of cerebral ischemia research, a chronic ischemia model using microcoils in the common carotid artery has been established; it displays the cognitive impairments associated with chronic cerebral ischemia [14]. Thus, in this study, we aimed to establish a chronic renal ischemia model using microcoils in the renal artery.

## 2 Materials and METHODS

### 2.1 Ethics

The experimental protocol was approved by the Research Center for Animal Life Science of Shiga University of Medical Science (Approval No. 2019-5-3). All animal experiments complied with the ARRIVE guidelines and were conducted in accordance with the National Institutes of Health Guide for the Care and Use of Laboratory Animals (NIH Publication No. 8023, revised 1978).

### 2.2 Animals

Eight-week-old male C57/BL6J mice were purchased from Jackson Laboratory (Bar Harbor, ME, USA). All mice were housed in a temperature-controlled environment (23 °C) within facilities operated by the Research Center for Animal Life Science at Shiga University of Medical Science. While in the center, mice underwent regular health examinations, including bacteriological tests. All mice were given free access to food and water.

### 2.3 Surgery

The procedure was performed with mice under adequate anesthesia via continuous isoflurane inhalation; a warming plate was used during the procedure to prevent hypothermia. Coiling and sham operations were performed for evaluation at 12 weeks (coiled group: n = 9, sham group: n = 6) and 5 days (coiled group: n = 11, sham group: n = 11) postoperatively. To prevent intravascular coagulation, mice were intraperitoneally administered 1000 units/kg of heparin 30 min before surgery. The surgical procedure is shown in Supplementary Movie 1. The bilateral kidneys were exposed through a midline abdominal incision. Next, fat around the right renal artery was removed; the right renal artery was exposed and freed. Two 6-0 silk threads were placed (one each on the distal and proximal sides of the renal artery) to gently lift the renal artery and place a microcoil around it. Subsequently, the abdominal cavity was washed with phosphate-buffered saline and the abdomen was closed. The procedure was completed when the mouse awakened from anesthesia and exhibited both independent movement and oral intake.

### 2.4 Microcoils

Microcoils were purchased from Samini Co., Ltd. (Shizuoka, Japan). The inner diameter and length of the microcoils were 0.26 mm and 2 mm, respectively.

### 2.5 Renal blood flow measurement

Renal blood flow was measured at the time of surgery and after 12 weeks of renal artery coiling. While mice were under anesthesia with continuous isoflurane inhalation, renal blood flow measurements were conducted using a laser blood flow measurement device (Omega Zone; Omegawave, Espoo, Finland). Five measurements were conducted in each kidney, and the mean value was recorded for analysis of renal blood flow.

### 2.6 Blood pressure and heart rate measurements

Blood pressure and heart rate were measured in awake mice using a programmable tail-cuff sphygmomanometer (BA98-A; Softron, Tokyo, Japan) [15]. Seven measurements were conducted in each mouse, and the mean values were recorded for analyses of blood pressure and heart rate. During the evaluation at 12 weeks postoperatively, blood pressure measurements were recorded for the coiled (n = 9) and sham (n = 6) groups. Similar measurements were recorded at 5 days postoperatively for the coiled (n = 11) and sham (n = 11) groups.

### 2.7 Blood and urinary analyses

Blood samples were collected from the left ventricle while the mice were under anesthesia with continuous isoflurane inhalation. Urine samples were collected over a 24-h period while mice were in metabolic cages with free access to food. Plasma cystatin C levels and urinary albumin were measured as previously described [16]. Urine KIM-1 levels were measured using a KIM-1 ELISA kit (R&D SYSTEMS, Minneapolis, MN, USA) according to the manufacturer's instructions. Other blood tests (urea nitrogen, creatinine, sodium, potassium, chloride, aspartate aminotransferase, and alanine aminotransferase) were conducted by OrientalBio Co., Ltd. (Tokyo, Japan).

### 2.8 Histological analyses

After 12 weeks of renal artery coiling, mice were perfused with phosphate-buffered saline under deep anesthesia following completion of renal blood flow assessment. The kidneys were removed and embedded in paraffin, then sliced into 3-μm-thick slices. Immunohistochemistry analyses were performed as previously described [17], using antibodies to F4/80 (Bio-Rad, Hercules, CA, USA) and fibronectin (Sigma-Aldrich, St. Louis, MO, USA).

### 2.9 Statistics

Data are presented as mean ± standard error of the mean. Analysis of variance followed by Tukey's *post hoc* test was used to identify significant differences among ≥3 groups; Student's *t*-test was used for comparisons between two groups. Prism 9 software (GraphPad, San Diego, CA, USA) was used for statistical analyses. *P*-values <0.05 were considered indicative of statistical significance.

## 3 Results

### 3.1 Renal artery coiling-based chronic hypoperfusion procedure

**Fig. 1:**
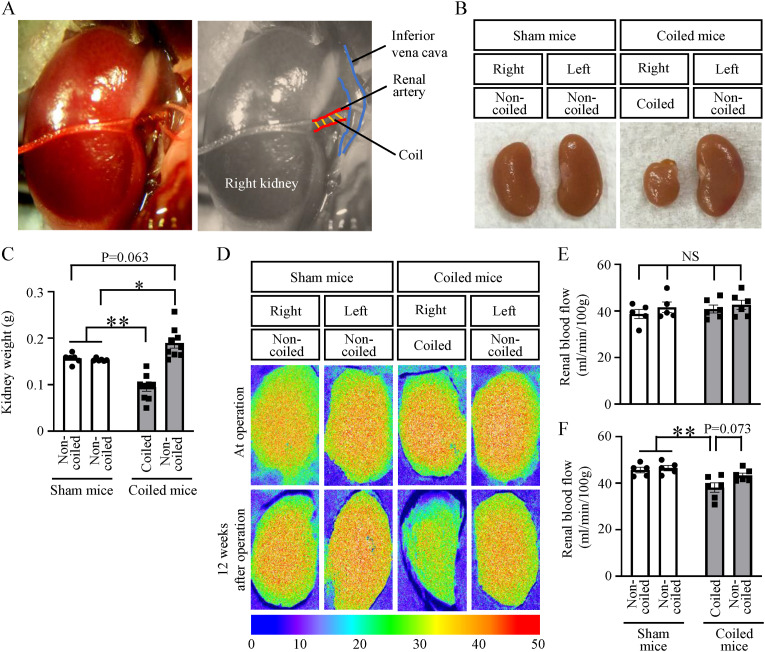
Establishment of the renal artery coiling-based chronic hypoperfusion model (A) Representative image of coiled renal artery. (B) Macroscopic renal morphology of coiled and non-coiled kidneys from sham-operated and coiled mice. (C) Kidney weights of coiled and non-coiled kidneys from sham-operated and coiled mice. (D) Representative images of renal blood flow at the time of surgery and at 12 weeks after surgery. (E, F) Renal blood flow levels in coiled and non-coiled kidneys from sham-operated and coiled mice at the time of surgery (E) and at 12 weeks after surgery (F). Data are shown as mean ± standard error of the mean. *p < 0.05, **p < 0.01, NS indicates no significance.

The surgical procedure is shown in Supplementary Movie 1. After the bilateral kidneys had been exposed through a midline abdominal incision, the right renal artery was exposed and freed. Two strands of 6–0 silk thread were placed (one each on the distal and proximal sides of the renal artery) to gently lift the renal artery and permit microcoiling (Fig. 1A).

### 3.2 Renal artery coiling-mediated changes in renal morphology and blood flow

Renal morphology, histology, and function were evaluated at 12 weeks after the coiling procedure. In the coiled group, kidneys on the coiled side showed morphological atrophy (Fig. 1B). Compared with kidney weights in the sham-operated group, kidney weights were significantly lower on the coiled side in the coiled group (Fig. 1C and Supplementary Fig. 1). In contrast, kidney weights on the non-coiled side in the coiled group were significantly greater, compared with kidney weights in the sham-operated group (Fig. 1C and Supplementary Fig. 1). There was no difference in renal blood flow between the sham and coiled groups at the time of surgery, but after 12 weeks of renal artery coiling, renal blood flow was significantly lower in the coiled kidneys than in the sham group (Fig. 1D–F). These results indicated that the renal artery coiling procedure caused chronic renal artery hypoperfusion leading to renal atrophy in coiled kidneys, along with compensatory renal hypertrophy in non-coiled kidneys.

### 3.3 Renal artery coiling-mediated decline in renal function

**Fig. 2:**
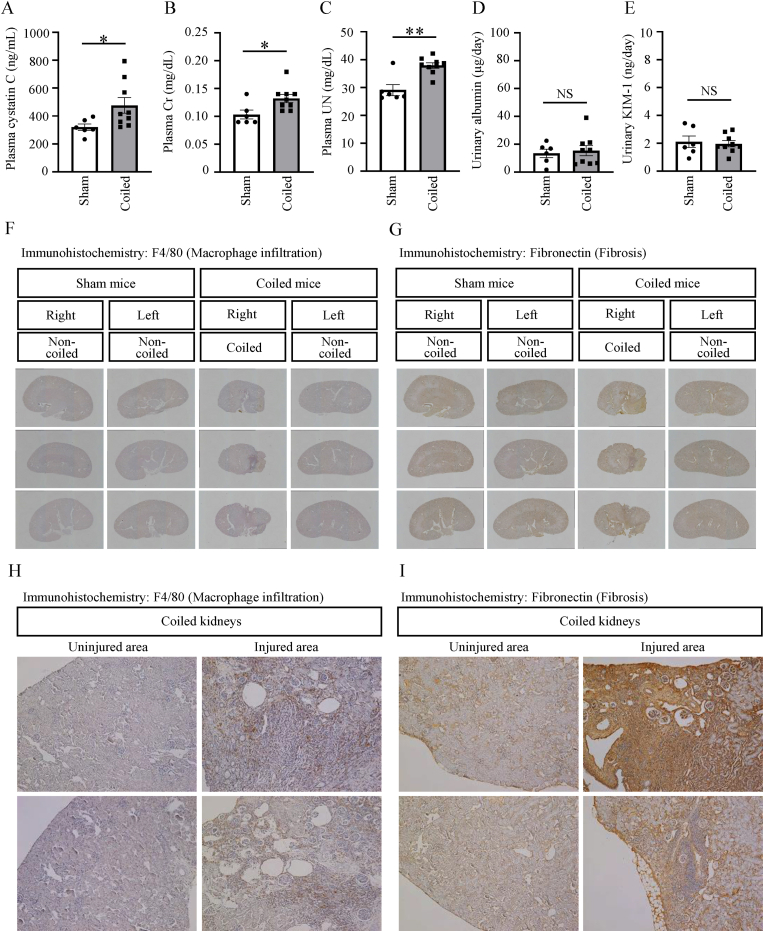
Renal injury in the established renal artery coiling-based chronic hypoperfusion model (A–C) Plasma cystatin C levels (A), plasma creatinine (Cr) levels (B), and plasma urea nitrogen (UN) levels (C) in sham-operated and coiled mice at 12 weeks after coiling. (D, E) Urinary albumin excretion levels (D) and KIM-1 levels (E) in sham-operated and coiled mice at 12 weeks after coiling. (F, G) Representative images showing immunohistochemistry analyses of F4/80-positive macrophage infiltration (F) and fibronectin deposition (G) in kidneys from sham-operated and coiled mice at 12 weeks after coiling. Original magnifications: × 40. (H, I) Representative images showing immunohistochemistry analyses of F4/80-positive macrophage infiltration (H) and fibronectin deposition (I) in uninjured and injured areas of kidneys from coiled mice. Original magnifications: × 100. Data are shown as mean ± standard error of the mean. *p < 0.05, **p < 0.01, NS indicates no significance.

Next, renal function was evaluated. Plasma levels of cystatin C, creatinine, and urea nitrogen were significantly greater in the coiled group than in the sham-operated group (Fig. 2A–C). However, urinary albumin and KIM-1 excretion did not differ between the two groups (Fig. 2D and E). As shown in Fig. 2F and G, there were local lesions with inflammation and fibrosis in kidneys on the coiled side; these manifestations were not present in kidneys on the non-coiled side in the coiled group or in any kidneys in the sham-operated group. The staining images of all kidneys are shown in Supplementary Figs. 2 and 3. The coiled kidneys displayed both injured and non-injured areas, with obvious cortical atrophy (Fig. 2H and I).

**Fig. 3:**
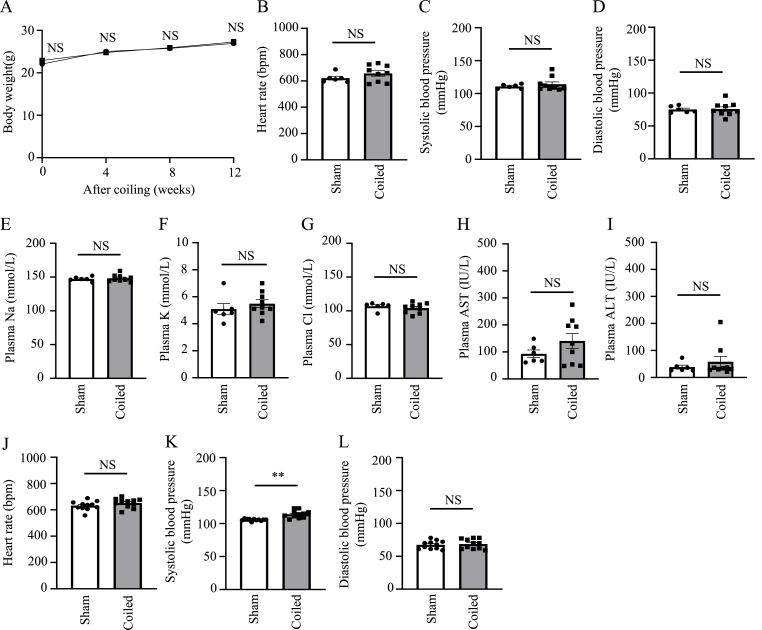
Changes in systemic conditions in the established renal artery coiling-based chronic hypoperfusion model (A) Changes in body weights of sham-operated and coiled mice during the experimental period. (B–D) Heart rate (B), systolic blood pressure (C), and diastolic blood pressure (D) in sham-operated and coiled mice at 12 weeks after coiling. (E–I) Plasma sodium (Na) (E), potassium (K) (F), chloride (Cl) (G), aspartate aminotransferase (AST) (H), and alanine transaminase (ALT) (I) in sham-operated and coiled mice at 12 weeks after coiling. (J–L) Heart rate (J), systolic blood pressure (K), and diastolic blood pressure (L) in sham-operated and coiled mice at 5 days after coiling. Data are shown as mean ± standard error of the mean. *p < 0.05, **p < 0.01, NS indicates no significance.

Finally, we evaluated systemic conditions. The growth characteristics and vital signs of the mice were observed to explore whether renal artery coiling affected any areas other than the kidneys. The body weights of 8-week-old male mice (20–25 g) did not differ between the sham-operated and coiled groups before surgery, and the rate of body weight gain was similar between the two groups until 12 weeks after coiling (Fig. 3A). Heart rate, systolic blood pressure, and diastolic blood pressure were also similar between the two groups at 12 weeks after surgery (Fig. 3B–D). Finally, there were no significant differences in plasma electrolytes (e.g., sodium, potassium, and chloride) or liver function between the sham-operated and coiled groups (Fig. 3E–I).

At 12 weeks postoperatively, there was no significant increase in blood pressure. However, we suspected that blood pressure was elevated in the early postoperative period. Therefore, we performed blood pressure measurements on the fifth day postoperatively. These measurements showed that systolic blood pressure was slightly elevated in a statistically significant manner on postoperative day 5 among mice in the coiled group, compared with mice in the sham group (Fig. 3J–L).

Collectively, the results showed that 12 weeks of unilateral renal artery coiling-induced chronic renal hypoperfusion led to a decline in renal function with focal renal tissue damage indicative of nephrosclerosis, without any other adverse effects on growth, vital signs, or electrolytes.

## 4 Discussion

In this study, we successfully developed a novel mouse model in which renal artery coiling induces chronic renal artery stenosis. This model can cause decreased blood flow in the kidney on the coiled side, along with focal fibrosis and atrophy in the renal parenchyma, and a decline in renal function. However, it does not affect blood pressure or other vital signs in the long term. Additionally, we did not detect any increase in the tubular damage marker KIM-1, which is typically present in cases of acute renal injury. Thus, our model is suitable for evaluating chronic renal ischemia caused by renal arteriosclerosis. Although there has been a report of coiling in the internal carotid artery to create a model of chronic cerebral ischemia [14], this is (to our knowledge) the first report of coiling in the renal artery.

The 2K1C (2-kidney, 1-clip) model has been described as a model of renal artery stenosis with similarities to our current approach [[18], [19], [20]]. This model exhibits hypertension accompanied by a sustained increase in renin activity and severe damage to the renal tubulointerstitium. In contrast, our coiling method produced a transient increase in blood pressure, potentially linked to a temporary increase in renin activity, but this change in blood pressure did not persist. Additionally, our model only caused focal fibrosis and atrophy. The disparate blood pressures and renal phenotypes between these two models may be attributed to differences in the severity of renal artery stenosis, although the underlying causes remain unclear. Nevertheless, we suggest that the 2K1C model is useful for elucidating the pathology of hypertension and renal injury associated with renal artery stenosis, whereas our model may be useful for efforts to understand ischemic renal injury in the absence of chronic hypertension.

Chronic ischemia has the potential to cause renal injury that results in ESKD, particularly among older patients with atherosclerotic renal artery stenosis [7,9]. To gain insight into the mechanisms of this injury, the pathology of the ischemic kidney must be explored [21]. Our novel model offers a potent tool for future exploration of the mechanisms of renal artery stenosis-related renal parenchymal injury. Renal hypoperfusion can lead to various changes such as atrophy, focal necrosis, epithelial regeneration, apoptosis, inflammation, interstitial fibrosis, and glomerulosclerosis [22]. According to our model, there exist regions that demonstrate various degrees of impairment, depending on the extent of hypoperfusion. In the affected regions, we observed atrophy (primarily cortical and tubular) and concomitant fibrosis, consistent with clinical findings in patients with renal artery hypoperfusion. Furthermore, this model can facilitate investigations regarding the effects of acquired factors (e.g., aging and obesity) on renal blood flow, as well as genetic factors (through the incorporation of transgenic/knockout mice) and drug administration on treatment outcomes.

This study had some limitations. First, the model only produces stenosis in the main trunk of the renal artery; it does not elicit atherosclerosis in the renal parenchyma, which is associated with hypertension and microangiopathy. Second, the study only included male mice; the effects of sex differences were not evaluated.

In conclusion, we established a mouse model of renal artery coiling-based chronic renal hypoperfusion. This model offers a powerful tool for future studies of renal arteriosclerosis-induced chronic renal ischemia.

## CRediT authorship contribution statement

**Yoshimi Imamura-Uehara:** Writing – original draft, Visualization, Validation, Methodology, Investigation, Formal analysis, Conceptualization. **Mako Yasuda-Yamahara:** Writing – original draft, Visualization, Validation, Methodology, Investigation, Formal analysis, Conceptualization. **Shogo Kuwagata:** Writing – original draft, Formal analysis. **Kosuke Yamahara:** Writing – original draft, Visualization, Formal analysis. **Mamoru Yoshibayashi:** Writing – original draft, Formal analysis. **Yuki Tanaka-Sasaki:** Formal analysis. **Akio Shimizu:** Data curation. **Hisakazu Ogita:** Data curation. **Masami Chin-Kanasaki:** Methodology, Formal analysis. **Shinji Kume:** Writing – original draft, Visualization, Supervision, Project administration, Methodology, Investigation, Funding acquisition, Formal analysis, Conceptualization.

## Declaration of competing interest

The authors declare the following financial interests/personal relationships which may be considered as potential competing interests:Shinji Kume reports financial support was provided by Nippon Boehringer Ingelheim Co Ltd. Shinji Kume reports financial support was provided by 10.13039/501100001691Japan Society for the Promotion of Science. If there are other authors, they declare that they have no known competing financial interests or personal relationships that could have appeared to influence the work reported in this paper.

## Data Availability

Data will be made available on request.
