# Supplementary material for: Establishment of a novel mouse model of renal artery coiling-based chronic hypoperfusion-related kidney injury
Source: Biochem Biophys Rep. 2023 Dec 15;37:101607. doi: 10.1016/j.bbrep.2023.101607 (PMC10764247; doi:10.1016/j.bbrep.2023.101607)
Supplement: Multimedia component 1 [file mmc1.pptx]

## Slide 1
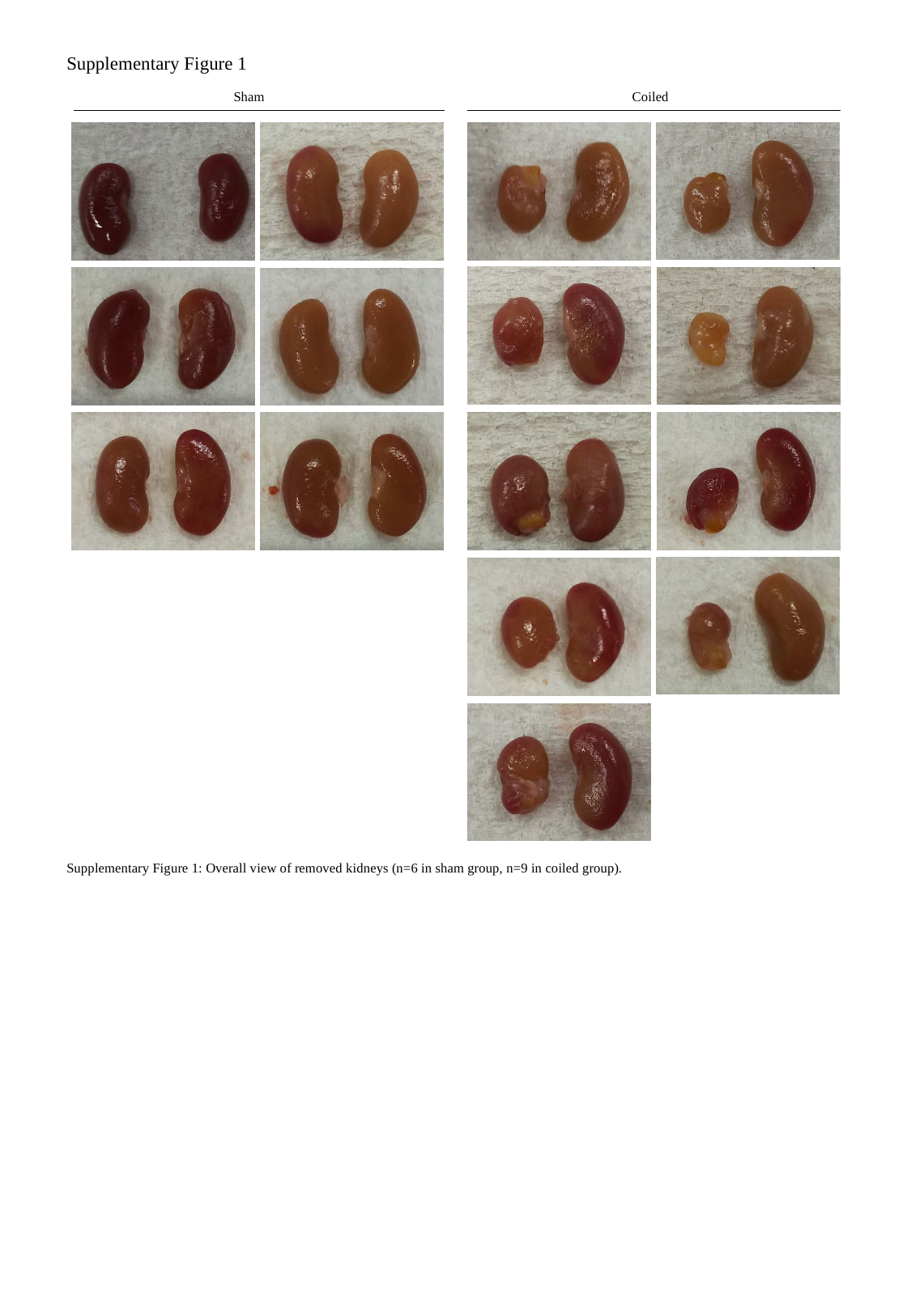

Supplementary Figure 1
Sham
Coiled
Supplementary Figure 1: Overall view of removed kidneys (n=6 in sham group, n=9 in coiled group).

## Slide 2
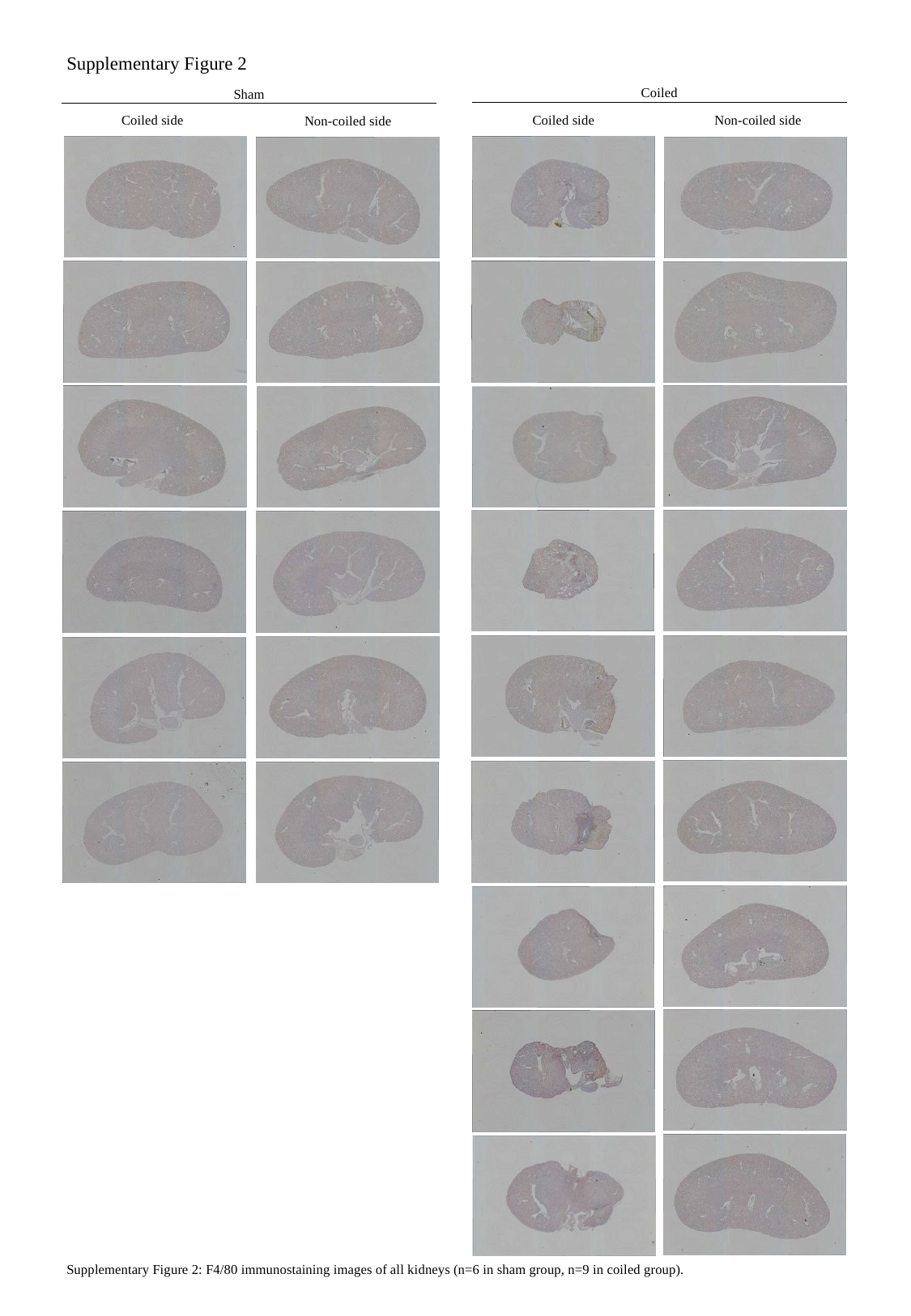

Supplementary Figure 2
Coiled
Sham
Coiled side
Coiled side
Non-coiled side
Non-coiled side
Supplementary Figure 2: F4/80 immunostaining images of all kidneys (n=6 in sham group, n=9 in coiled group).

## Slide 3
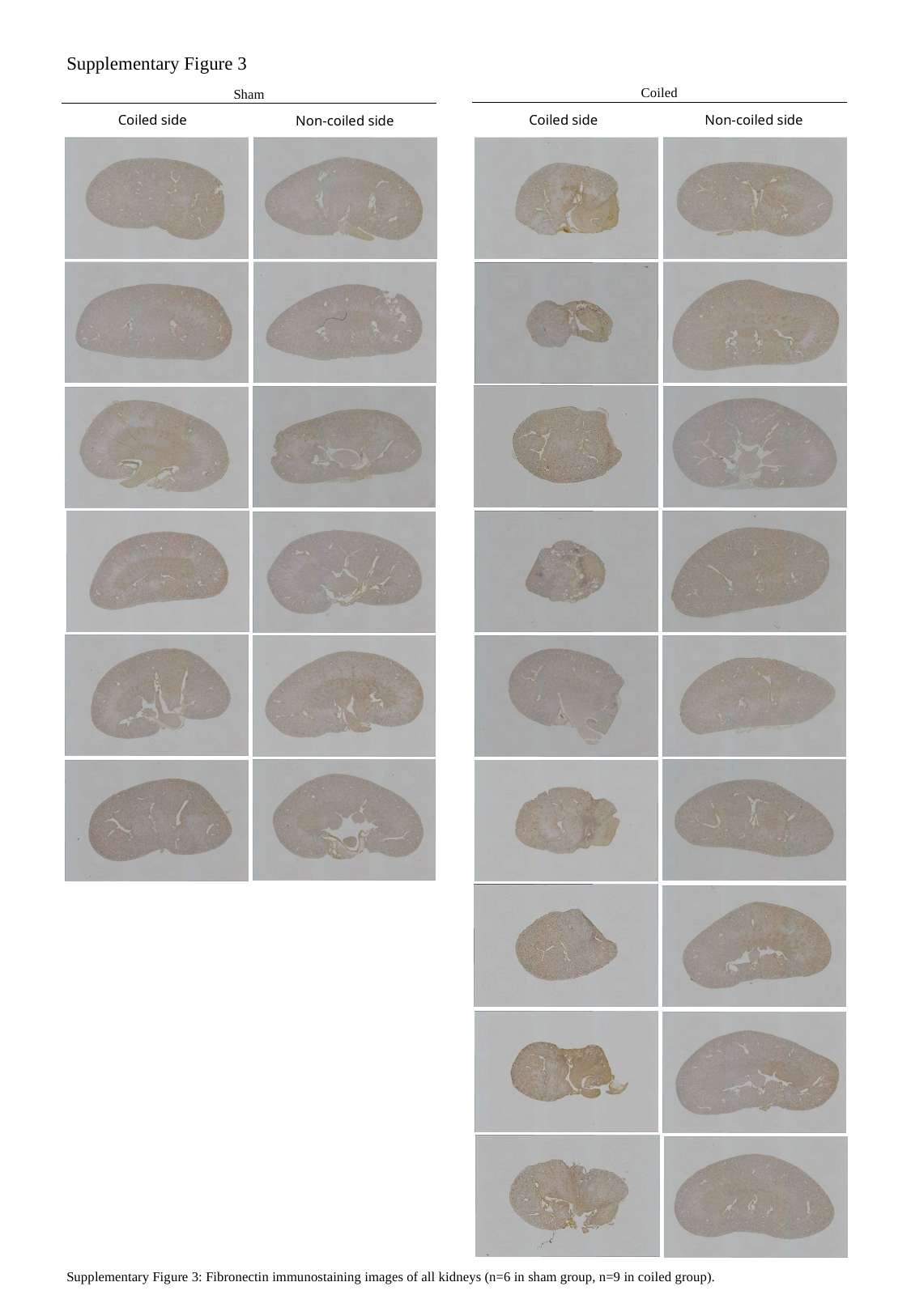

Supplementary Figure 3
Coiled
Sham
Coiled side
Coiled side
Non-coiled side
Non-coiled side
Supplementary Figure 3: Fibronectin immunostaining images of all kidneys (n=6 in sham group, n=9 in coiled group).
